# Host-specific functional evolution of seal influenza A virus NS1 protein following avian-to-seal transmission

**DOI:** 10.1128/jvi.01650-25

**Published:** 2026-03-24

**Authors:** Maryna Kuryshko, Christine Luttermann, Mahmoud Bayoumi, Ahmed Mostafa, Jula Weißmann, Alexander Schäfer, Lisa Wendt, Thomas Hoenen, Jendrik Müller, Luis Martinez-Sobrido, Thomas C. Mettenleiter, Elsayed M. Abdelwhab

**Affiliations:** 1 Institute of Molecular Virology and Cell Biology, Friedrich-Loeffler-Institut, Federal Research Institute for Animal Health 39023 , Greifswald-Insel Riems, Germany; 2 Institute of Immunology, Friedrich-Loeffler-Institut, Federal Research Institute for Animal Health 39023 , Greifswald-Insel Riems, Germany; 3 Disease Intervention and Prevention, Texas Biomedical Research Institute 7075, San Antonio, Texas, USA; 4 Virology Department, Faculty of Veterinary Medicine, Cairo University 110151 , Giza, Egypt; 5 Center of Scientific Excellence for Influenza Viruses, National Research Centre 68787, Giza, Egypt; 6 Institute of Diagnostic Virology, Friedrich-Loeffler-Institut, Federal Research Institute for Animal Health 39023 , Greifswald-Insel Riems, Germany; 7 Friedrich-Loeffler-Institut, Federal Research Institute for Animal Health 39023 , Greifswald-Insel Riems, Germany; University Medical Center Freiburg, Freiburg, Germany

**Keywords:** avian influenza virus (AIV), seals, cross-species transmission, NS1 protein, interferon antagonism, viral adaptation, zoonotic potential

## Abstract

Marine mammals, particularly seals, are susceptible to both avian and human influenza A viruses (IAVs), making them potential intermediates for zoonotic virus emergence. In recent decades, repeated transmissions of avian influenza viruses (AIVs) from wild aquatic birds, their natural reservoir, have caused significant mortality in seals. Defining the molecular determinants of viral adaptation in marine mammals, and their implications for replication in human cells, is therefore essential. The non-structural protein 1 (NS1) of AIV, a key antagonist of the interferon (IFN) response, plays a central role in host adaptation. Here, we analyzed NS1 proteins from seal influenza viruses (H3, H4, H5, H7, and H10 subtypes) and their closest avian relatives isolated between 1980 and 2023, and evaluated their function in seal, avian, and human cells. Phylogenetic analysis confirmed multiple bird-to-seal transmission events. Seal-derived NS1 proteins generally contained few strain-specific amino acid substitutions and showed comparable expression and IFN antagonism to their avian precursors. A notable exception was the seal H10N7 virus isolated in 2014 in Northeastern Europe, which harbored three previously uncharacterized substitutions at NS1 amino acid residues 94, 104, and 171. These amino acid substitutions markedly altered NS1 properties to enhance protein stability, suppress IFN induction, mediate host transcription shut-off, and increase polymerase activity in human cells, without affecting NS1 expression or reducing virus replication in avian cells. Overall, these results reveal how NS1 undergoes host-specific functional evolution following avian-to-seal transmission and provide mechanistic insight into the adaptation of influenza A viruses to mammalian hosts.

**IMPORTANCE:**

Avian influenza viruses (AIVs) circulate naturally in wild aquatic birds but occasionally infect mammals, including seals, where they can cause severe outbreaks. Seals are of particular concern because they can harbor both avian and human influenza viruses, creating opportunities for reassortment and the emergence of novel zoonotic strains. Understanding how AIVs adapt to mammalian hosts is therefore critical for anticipating and mitigating future influenza threats. Here, we investigated the role of the NS1 protein, a key viral factor that suppresses host immune responses, in seal-derived AIVs. Overall, NS1 expression and function were conserved across different subtypes and host cells. However, we identified unique amino acid substitutions in the NS1 of a seal H10N7 virus that enhanced protein stability, interferon antagonism, and viral adaptation in human cells. These findings illustrate how minor changes in NS1 protein can drive host adaptation and underscore the need for continued surveillance of AIVs in seals.

## INTRODUCTION

The global spread of avian influenza viruses (AIVs), exemplified by the ongoing H5N1 clade 2.3.4.4b panzootic, represents a major threat to animal and human health, wildlife, and biodiversity. AIVs, members of the genus influenza A virus (IAV) within the family *Orthomyxoviridae*, are classified based on their surface glycoproteins hemagglutinin (HA) and neuraminidase (NA), with 17 HA (H1 to H16 and H19) and 9 NA (N1 to N9) subtypes forming diverse HxNy combinations (1, 2). Two main pathotypes exist: low pathogenicity AIVs (LPAIVs), causing mild or asymptomatic infections, and high pathogenicity AIVs (HPAIVs), which induce severe disease and high poultry mortality. While all AIVs initially circulate as LPAIVs, certain H5 and H7 subtypes can evolve into HPAIVs (3). Wild waterfowl serve as the primary reservoir, but spillover into poultry and occasionally mammals, including humans, occurs, with most infections linked to direct bird contact (1).

Historically, pigs have been important intermediate mammalian hosts for AIV adaptation to humans. However, frequent infections in seals, cats, minks, and foxes highlight their potential role as intermediates in cross-species transmission. Seal respiratory tissues express both α2,3- and α2,6-linked sialic acid receptors, making them susceptible to avian and human influenza viruses (4–7) and potential hosts for the emergence of zoonotic influenza (8–11). Seal outbreaks have involved LPAIV (H1N1, H3N8, H4N5, H7N7, H10N4, and H10N7) and HPAIV (H5N8 and H5N1) subtypes, with evidence of seal-to-seal transmission and occasional evolution into mammal-specific clades (12–19). Human infection from seal-derived viruses, including H7N7 conjunctivitis in Massachusetts in 1979, has been reported (20). Recent studies suggest that human-adaptive amino acid substitutions can emerge in non-human mammals (21–24), underscoring the potential of seals as intermediate hosts and increasing the risk of zoonotic influenza emergence. Indeed, some seal-adapted AIVs can acquire amino acid substitutions that enhance human receptor binding or replication in mammals, sometimes without affecting pathogenicity in birds (11, 14, 25–30). Although direct seal-human contact is generally less frequent than bird-to-human exposure, occupational exposure, ecotourism, or interactions with seals in rehabilitation centers, aquariums, research facilities, coastal fishing operations, or during wildlife rescue and monitoring activities could provide opportunities for cross-species transmission (31).

Animal to human transmission of AIV has traditionally been associated with amino acid substitutions in the HA protein and the viral polymerase complex (32, 33). However, the recent transmission of clade 2.3.4.4b H5N1 in the United States occurred without a significant shift in HA receptor-binding affinity (34), suggesting that HA-independent genetic factors may also facilitate cross-species transmission (33). One such factor is the non-structural protein 1 (NS1), a key viral antagonist of the host immune response that contributes to both virulence and replication of IAV in humans (35). Notably, NS1 amino acid substitutions were among the genetic changes that accompanied the avian-to-human adaptation of the 1918 pandemic H1N1 strain (36). The NS1 protein from the 1918 influenza virus was shown to more effectively suppress interferon (IFN) responses in human cells than NS1 proteins from seasonal or mouse-adapted strains (37).

NS1 is a multifunctional, 230-amino-acid protein composed of an RNA-binding domain (RBD; residues 1–73) and an effector domain (ED; residues 86–230), linked by a flexible connector (residues 74–85) (38). The RBD mediates interactions with RNA, while the ED binds host proteins such as cleavage and polyadenylation specificity factor 30 (CPSF30), Protein Kinase R (PKR), and polyadenylate-binding protein II (PABPII), thereby inhibiting IFN responses and supporting viral replication before host immunity is activated (39, 40). Based on sequence analysis, NS1 genes are classified into two major alleles: A and B, which differ by more than 30% in amino acid sequence and are further subdivided into Eurasian and American lineages (41, 42). Allele A predominates in avian hosts. In avian cells, viruses encoding either allele replicate with variable efficiency, but both similarly modulate type I IFN responses (43). In mammalian systems, allele B was initially regarded as less adapted, whereas allele A more effectively suppressed IFN-β expression in human and mink lung cells (44, 45). More recent findings, however, show that both alleles can replicate efficiently in mammals (46) and that specific amino acid substitutions in NS1, independent of allele, enhance replication, immune evasion, and host adaptation (47, 48).

Despite these findings, the functional role of adaptive amino acid substitutions in seal-derived NS1 remains poorly understood, particularly in the context of immune evasion and host adaptation in non-human mammals such as seals. Further investigation is needed to elucidate the contribution of NS1 to AIV adaptation in these emerging mammalian hosts. Here, we show that the NS1 proteins from seal influenza viruses generally resembled their avian precursors in expression and IFN antagonism. However, the seal H10N7 virus from 2014 carried three unique amino acid substitutions (at residues 94, 104, and 171) that enhanced NS1 stability, IFN suppression, host transcription shutoff, and polymerase activity in human cells, without impairing replication in avian cells.

## RESULTS

### Strain-specific NS1 changes in some seal influenza viruses

**Fig 1:**
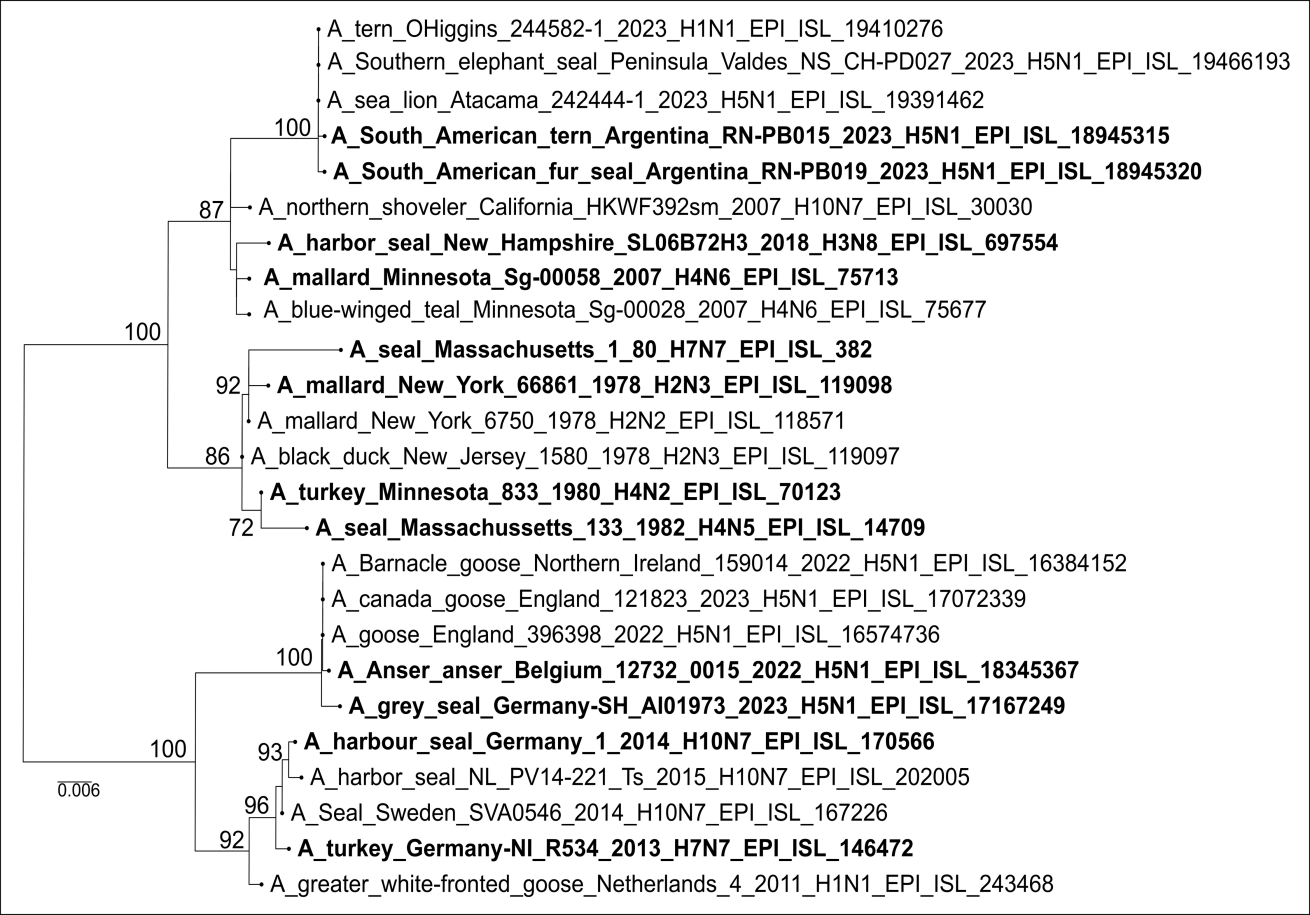
Phylogenetic analysis of NS segment of seal IAV. NS gene sequences of seal and AIVs were retrieved from GISAID and aligned using MAFFT. Midpoint-rooted phylogenetic trees were constructed with IQ-TREE, and representative viruses were selected to generate the current tree using phylogeny.fr and further edited using FigTree.

To examine the origin and relatedness of NS1 genes in seal influenza viruses, we conducted a phylogenetic analysis of all seal-derived NS sequences available in GISAID as of 18 September 2025 (Fig. 1). Seal AIVs of subtypes H1, H3, H4, H5, H7, and H10 were identified. Phylogenetic and sequence comparisons of H3 to H10 IAV indicated that all seal NS1 genes originated from avian ancestors, supporting bird-to-seal transmission as the primary route of introduction. Strikingly, all viruses carried allele A, except for A/Gray seal/England/2017(H3N8) (24), which clustered with allele B.

Representative NS1 sequences from seal AIVs (H3N8, H4N5, H5N1 clade 2.3.4.4b, H7N7, and H10N7; 1980–2023) were compared to their closest avian ancestors (Table 1). The historic A/Seal/Massachusetts/1/80 (H7N7) exhibited the highest number of changes (I68M, E70K, I129M, G168R, V178I, and R204S), followed by A/harbor seal/Germany/1/2014 H10N7 with three substitutions, while H5N1/2023 clade 2.3.4.4b isolates showed one to two substitutions depending on the strain (Table 1). Most amino acid substitutions were located in the effector domain. All amino acid substitutions were strain specific, except for residue 178, which was altered in two viruses: I178V in A/seal/Massachusetts/133/1982 H4N5 and V178I in A/Seal/Massachusetts/1/80 H7N7. These findings suggest that strain-specific NS1 amino acid substitutions acquired in seals may contribute to viral adaptation in mammals.

**TABLE 1:** Seal viruses and closest avian ancestors selected in this study

Subtype	Seal virus	Closest IAV ancestor	Abbrev. (seal/avian)	NS1 amino acid substitution	
				Nucleotide	Amino acid
H3N8	A/harbor seal/New Hampshire/SL06B72H3/2018	A/mallard/Minnesota/Sg-00058/2007 H4N6	seH3N8/avH4N6	7	R88H
H4N5	A/seal/Massachusetts/133/1982	A/turkey/Minnesota/833/80 H4N2	seH4N5/avH4N2	5	**I178V**
H5N1	A/gray seal/Germany-SH/AI01973/2023	A/Anser anser/Belgium/12732_0015/2022 H5N1	seH5N1_1/avH5N1	2	*A76V* and I123V
H5N1	A/South American fur seal/Argentina/RN-PB019/2023	A/South American tern/Argentina/RN-PB015/2023 H5N1	seH5N1_2/avH5N1_1	1	*E55K*
H7N7	A/Seal/Massachusetts/1/80	A/mallard/New_York/66861/1978 H2N3	seH7N7/avH2N3	11	*I68M*, *E70K*, I129M, G168R, **V178I**, and R204S
H10N7^*a*^	A/harbor seal/Germany/1/2014	A/turkey/Germany-NI/R534/2013 H7N7	Seal/avian	5	T94I, T104M, and D171Y

^*a*^ H10N7 has been detected in seals in Germany, Sweden, Netherlands, and Denmark. Residues written in italics are in the RNA binding or linker domains; position 178 was seen twice and is written in bold.

### Expression levels upon transfection of NS1 in different host backgrounds are not affected by the avian-to-seal sequence mutations

**Fig 2:**
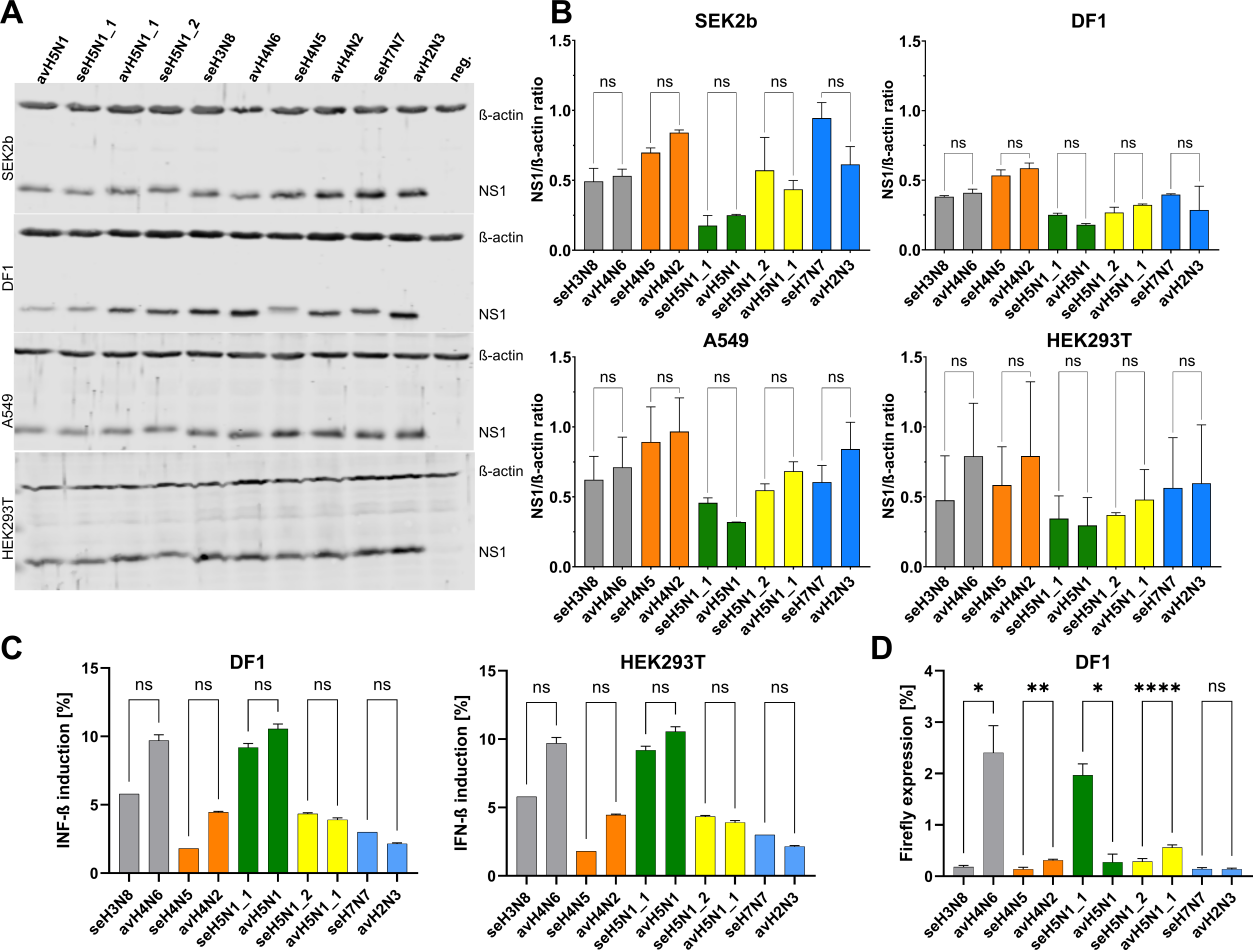
NS1 expression and IFN-β suppression in seal, avian, and human cells. NS1 protein expression was analyzed in seal SEK2b, avian DF1, and human A549 and HEK293T transfected cells. Cells were transfected with 2 µg of pcDNA-NS1 plasmid and incubated at 37°C with 5% CO_2_ for 24 h. Proteins were extracted, resolved on 10% SDS-PAGE, and transferred to nitrocellulose membranes. Membranes were probed with rabbit anti-NS1 and monoclonal anti-β-actin antibodies, followed by IRDye 680/800CW secondary antibodies. Signals were detected using an Odyssey Clx Imager. Representative blots are shown in panel **A**. Experiments were performed in duplicate and independently repeated three times. Band intensities were quantified using Image Studio Lite 5.2, and data are expressed as mean ± SD of the NS1/β-actin ratio. pcDNA3 empty plasmid transfected cells served as controls for normalization (**B**). NS1-mediated inhibition of IFN-β induction was assessed in DF1 and HEK293T cells pre-activated with MDA5 using IFN reporter assay (**C**). DF1 cells were co-transfected with plasmids encoding wild-type or mutant NS1 proteins (1 µg/well) together with pCAGGS Firefly luciferase construct (0.25 µg/well) using Lipofectamine 2000. After 20 h, luciferase activity was quantified. The assay was performed in three independent biological replicates (**D**). Asterisks denote statistical significance (****P < 0.0001) compared with the avian NS1 ancestor. NC, negative control cells without NS1 transfection. SEK2b, harbor seal kidney cells; DF1, chicken fibroblast cell line; A549, human lung adenocarcinoma cell line; HEK293T, human embryonic kidney 293T cell line; NC, negative control; NS1, non-structural protein 1; IFN, interferon; MDA5, melanoma differentiation-associated protein 5; neg., non-transfected cells. H/N subtypes in this figure correspond to the bold-printed subtypes in the phylogenetic tree shown in Fig. 1A and the entries listed in Table 1. Asterisks indicate statistical significance (*P < 0.05, **P < 0.01, and ****P < 0.0001) compared to the avian ancestor; ns, not significant.

To assess whether amino acid substitutions in NS1 affect its expression, coding sequences from seal H3N8, H4N5, H5N1, H7N7, and H10N7 viruses, along with their avian progenitors, were cloned into the pCDNA3 expression vector. These constructs were transfected into seal kidney (SEK2b), chicken fibroblast (DF1), and human (HEK293T and A549) cell lines. NS1 protein levels were detected at 24 h post-transfection using a polyclonal antibody and standard Western blotting techniques. Expression was quantified and normalized to housekeeping proteins (Fig. 2A and B). Minor variations in NS1 expression were observed among the different cell types; however, these differences were not statistically significant. These findings suggest that sequence variation in NS1 does not markedly impact its expression level across seal, avian, and human hosts.

### Conserved IFN suppression by seal NS1 with strain-specific effects on reporter expression

We next evaluated the ability of NS1 from seal IAVs to suppress IFN induction in avian and human cell lines using IFN reporter assay (47, 49). Due to the lack of established data on seal IFN responses, attempts to develop this assay in seal cells were inconclusive. Results showed that NS1 proteins from seal H3N8, H4N5, H5N1, and H7N7 viruses, as well as their respective avian ancestors, were similarly effective in suppressing IFN-β induction in both chicken and human cells (Fig. 2C). To explore potential effects beyond IFN signaling, we assessed NS1-mediated suppression of firefly luciferase (FFL) expressed from a constitutive CAG promoter in DF1 cells (Fig. 2D). While IFN suppression was similar across all NS1 proteins, NS1 from seal H3N8, H4N5, and H5N1 (A/South American fur seal/Argentina/RN-PB019/2023; seH5N1_2, Table 1) significantly reduced luciferase expression compared to their avian counterparts, whereas NS1 from seal H7N7 and A/gray seal/Germany-SH/AI01973/2023 H5N1 (seH5N1_1) showed similar or slightly weaker effects, respectively. Because luciferase expression measures protein output, these differences may reflect effects at the transcriptional or translational level. These results indicate that strain-specific differences in NS1 variants can modulate reporter expression, but IFN antagonism remains largely conserved.

### Fine-tuning of H10N7 NS1 expression by effector domain substitutions T94I, T104M, and D171Y

**Fig 3:**
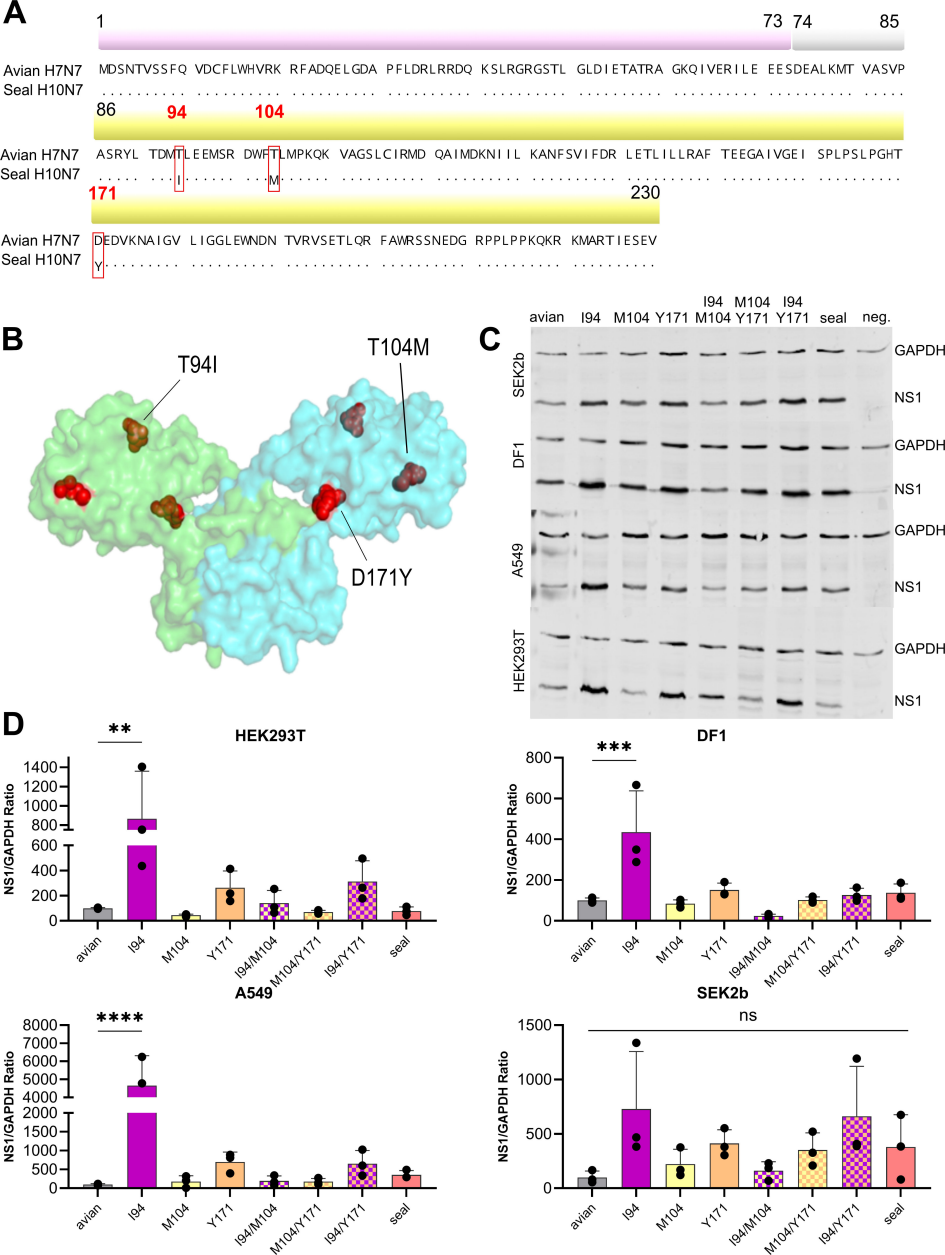
Impact of amino acid substitutions on H10N7 NS1 protein expression in seal, avian, and human cells. Seal-derived H10N7 NS1 harbored unique amino acid substitutions (residues 94, 104, and 171), highlighted in the sequence alignment in the effector domain (yellow bar) (**A**) and mapped as red spheres on the predicted NS1 dimer structure. The tertiary structure was modeled using SWISS-MODEL and edited by Inkscape (**B**). Seal, human, and avian cells were transfected with pCAGGS plasmids encoding NS1 variants, including the avian ancestor, seal-derived NS1, and avian NS1 with single or double amino acid substitutions at positions 94, 104, and 171. After 24 h, cell lysates were subjected to near-infrared (NIR) Western blotting (**C**), and protein levels were normalized to GAPDH (**D**). Asterisks indicate statistical significance (**P < 0.01, ***P < 0.001, and ****P < 0.0001) compared with the avian NS1 ancestor (avian); ns, not significant. SEK2b, harbor seal kidney cells; DF1, chicken fibroblast cell line; A549, human lung adenocarcinoma cell line; HEK293T, human embryonic kidney 293T cell line; neg., non-transfected cells; seal, H10 N7 NS1 with triple mutations; avian, H7 N7 NS1.

NS1 from seal H10N7, associated with the 2014 European outbreak that killed ~10% of the wild seal population (12), displayed a distinct functional profile. It carries three unique substitutions in the effector domain (T94I, T104M, and D171Y) compared to its avian ancestor (Fig. 3A and B). Similar to NS1 proteins from seal H3N8, H4N5, H5N1, and H7N7, expression levels of H10N7 NS1 across seal, chicken, and human cells were comparable to the avian NS1 (Fig. 3C and D). To determine whether the three H10N7 NS1 substitutions act independently or synergistically to modulate NS1 expression, plasmids encoding wild-type and mutant NS1 proteins were transfected into chicken, human, and seal cells (Fig. 3C and D). The results were striking: the T94I substitution alone significantly increased NS1 expression in avian and human cells (*P* < 0.01), and a similar trend was observed in seal cells, although it failed to reach statistical significance (*P* < 0.059). In contrast, the T104M and D171Y amino acid substitutions counteracted this increase, restoring expression to more balanced levels. These results indicate that residue I94 can enhance H10N7 NS1 expression, while M104 and Y171 modulate it, contributing to balanced NS1 levels across multiple host species.

### Seal H10N7 NS1 amino acid substitutions increase protein stability, driven mainly by residue M104 or Y171

**Fig 4:**
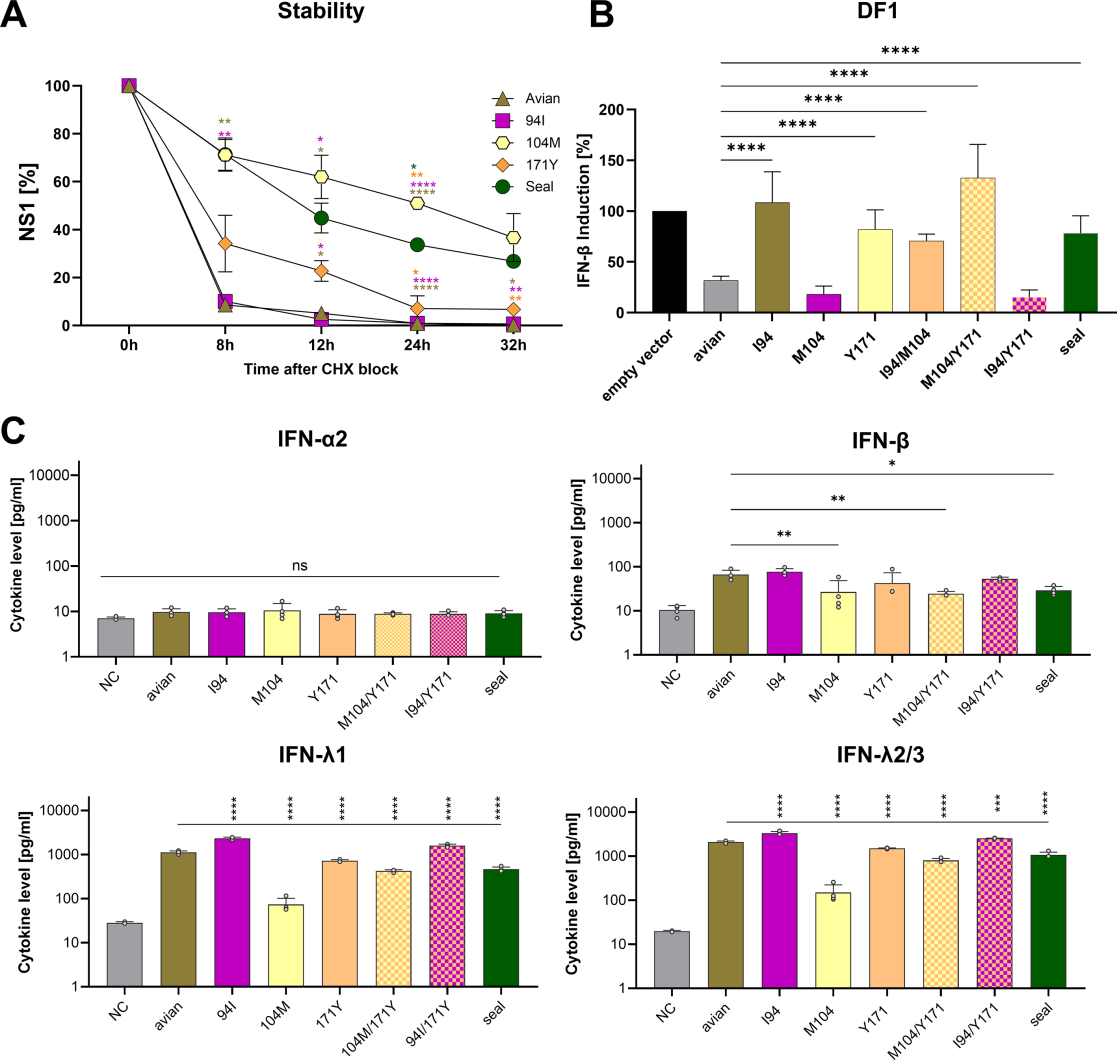
Impact of amino acid substitutions on H10N7 NS1 protein stability and interferon antagonism. The impact of specific amino acid substitutions in the NS1 protein of the seal-derived H10N7 virus on its stability. MDCK II cells were infected with reverse-genetically generated viruses at an MOI of 0.01 for 1 h in the presence of trypsin. Cycloheximide (CHX) was added at 24 h post-infection to a final concentration of 100 µg/mL to inhibit protein synthesis. Cells were harvested at 0, 8, 12, 24, and 32 h after CHX addition, and samples were analyzed by Western blot. Protein levels were quantified using the Odyssey Clx Imager and Image Studio Software to calculate the half-life of NS1 (**A**). Relative IFN induction inhibition in DF1 cells was performed using IFN reporter assay triggered with chicken MDA5 for 24 h (**B**). A549 cells were infected with recombinant viruses expressing avian NS1, seal-derived NS1, or avian NS1 variants carrying single or combined amino acid substitutions at residues 104 and 171 for 24 h. Interferon-β production was induced by adding poly(I:C) to the cells for an additional 24 h. Supernatants were collected and analyzed using the LEGENDplex Human Anti-Virus Response Panel 1. Data represent the mean ± SEM of three independent experiments, highlighting the critical role of these amino acid substitutions in the increased efficiency of seal NS1 in blocking IFN-β induction (**C**). Asterisks indicate statistical significance (*P < 0.05, **P < 0.01, ***P < 0.001, and ****P < 0.0001) compared to the avian ancestor; ns, not significant. NC refers to non-infected cells as negative controls. Amino acid substitutions were introduced into the avian H7N7 NS1 to match the seal NS1 sequence. MDCK II, Madin-Darby canine kidney type II cells; CHX, cycloheximide; A549, human lung adenocarcinoma cell line; DF1, chicken fibroblast cell line; IFN, interferon; NC, non-infected cells; seal, H10N7 NS1 with triple mutations; avian, H7N7 NS1; MOI, multiplicity of infection.

We next asked whether the observed differences in NS1 expression were linked to altered protein stability (Fig. 4A). To address this, we generated recombinant avian viruses carrying seal-derived NS1 or individual amino acid substitutions of NS1. When we amplified and sequenced segments of our egg-propagated seal virus and compared all gene segments to the corresponding GISAID sequences from the original seal sample, we identified multiple amino acid substitutions, including 10 amino acid differences in HA. We then reversed all amino acid substitutions across all gene segments to match the GISAID sequences and attempted to rescue the wild-type virus; however, this was unsuccessful. Consequently, the closest ancestral A/turkey/Germany/R534/2013 H7N7 virus was used as a surrogate backbone. Using this system, we introduced single, double, or triple amino acid substitutions into the H7N7 NS1 to mimic the seal NS1 sequence. Attempts to generate a virus carrying both T94I and T104M amino acid substitutions also failed, suggesting a detrimental effect of this combination.

MDCK II cells were infected with the recombinant viruses, and protein synthesis was inhibited with cycloheximide (CHX) 24 h post-infection. NS1 decay was monitored by Western blotting at 0, 8, 12, 24, and 32 h (Fig. 4A). Avian NS1 displayed a short half-life of 2.3 h. Substitution at residue T94I had no significant effect (*P* = 0.42), while T104M markedly extended half-life to 24.7 h (*P* < 0.000001, ~11-fold increase). The D171Y substitution also increased stability, extending the half-life to 5.6 h (*P* < 0.01), corresponding to an ~2.5-fold increase. Seal NS1 carrying all three substitutions exhibited an intermediate half-life of 15.3 h (~7-fold longer than avian NS1, *P* < 0.0001). These findings identify residue T104M as the main driver of enhanced NS1 stability, with residue D171Y providing additional stabilizing effects.

### Seal H10N7 NS1 amino acid substitutions enhanced IFN antagonism in human cells but reduced blocking in avian cells, driven mainly by residues 104 and 171

To investigate how seal-derived NS1 amino acid substitutions affect IFN inhibition, we measured type I and III IFN levels in human A549 cells (Fig. 4B) and assessed IFN-β promoter activity in avian DF1 cells (Fig. 4C) (47). In human cells, seal NS1 more efficiently suppressed IFN-β and IFN-λ than its avian ancestor (*P* < 0.05), whereas IFN-α remained unaffected. Introduction of M104, alone or together with Y171, further enhanced avian NS1-mediated suppression, particularly for IFN-λ (*P* < 0.001). Conversely, the I94 substitution reduced IFN inhibition (*P* < 0.01). In DF1 cells, avian NS1 was generally more effective at blocking IFN-β, but the presence of I94 or Y171 decreased this efficiency, and the double mutant I94/Y171 abolished the effects of individual substitutions. These findings indicate that NS1 amino acid substitutions acquired after transmission of H10N7 to seals enhance IFN-antagonism in human cells while reducing it in avian cells. Residue M104 is the primary driver of this host-specific, pleiotropic effect, with Y171 contributing to a lesser extent, whereas I94 consistently diminishes NS1-mediated IFN suppression across hosts.

### Amino acid substitution I94 modulates NS1 effects on host gene expression

**Fig 5:**
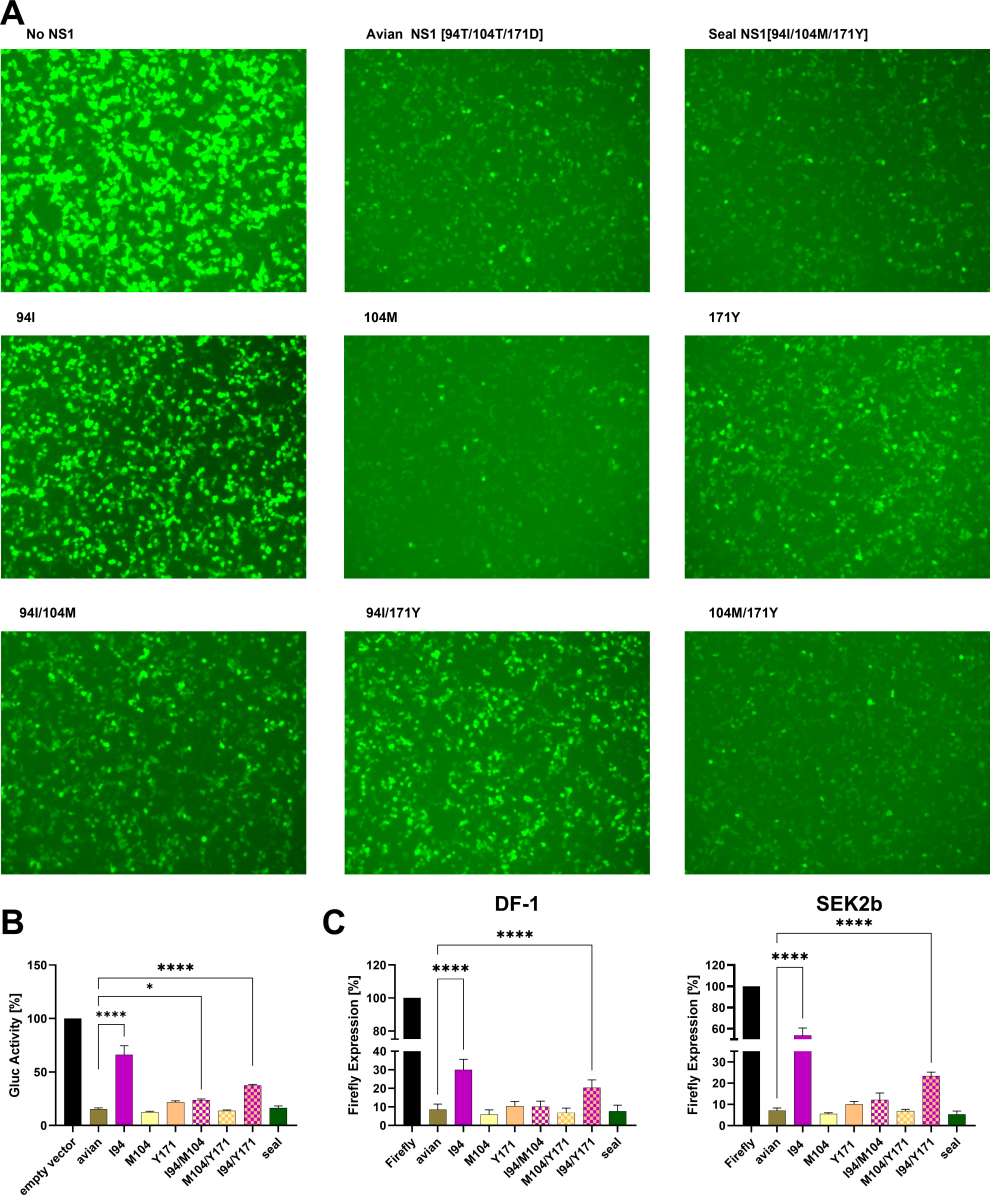
Seal NS1 amino acid substitutions modulate host transcription shutoff. HEK293T cells were co-transfected with wild-type or mutant NS1 plasmids together with Gaussia luciferase (Gluc) and GFP reporters. GFP expression was visualized by fluorescence microscopy (**A**), and Gluc activity in supernatants was quantified to assess the impact of NS1 amino acid substitutions on host gene expression. The measurements are shown as a mean with SD, and Gluc activity of the empty vector is set to 100% (**B**). DF1 and SEK-2b cells were co-transfected with plasmids encoding wild-type or mutant NS1 proteins (2 µg/well) together with pCAGGS Firefly luciferase constructs (0.25 µg/well) using Lipofectamine 2000. After 20 h, luciferase activity was quantified. The assay was performed in three independent biological replicates. The measurements are shown as a mean with SD, Firefly expression of the empty vector is set to 100% (**C**). Asterisks denote statistical significance (*P = 0.0233 and ****P < 0.0001) compared with the avian NS1 ancestor. NC, negative control cells without NS1 transfection. Amino acid substitutions were introduced into H7N7 NS1 to match the seal NS1 sequence. HEK293T, human embryonic kidney 293T cells; DF1, chicken fibroblast cell line; SEK2b, harbor seal kidney cells; NS1, non-structural protein 1; Gluc, Gaussia luciferase; GFP, green fluorescent protein; NC, negative control.

To clarify the mechanism underlying the host-specific differences in IFN antagonism, we asked whether seal NS1 amino acid substitutions affect global host gene expression or act specifically on IFN pathways. Human cells were co-transfected with plasmids encoding wild-type or mutant NS1 proteins together with green fluorescent protein (GFP) reporters. After 24 h, GFP expression was visualized by fluorescence microscopy (Fig. 4A). Both wild-type avian and seal NS1 suppressed reporter expression to comparable levels, consistent with broad inhibition of host gene expression. Strikingly, substitution I94 alone abolished this suppression, whereas M104 and Y171, alone or combined, preserved the inhibitory function of avian NS1 (Fig. 5A and B). The same pattern was observed in avian DF1 and seal SEK cells: wild-type NS1 proteins suppressed reporter expression, while I94, alone or with Y171, impaired this activity; M104 had no effect (Fig. 5C). Trials to study the potential interaction of NS1 with host factors essential for host transcription (i.e., CPSF30 and eIF4G) failed. Because GFP expression and reporter assays measure protein output, we cannot distinguish whether NS1 acts at the transcriptional or translational level. Nevertheless, these findings indicate that I94T is critical for NS1-mediated inhibition of host gene expression, independent of host origin, providing a mechanistic explanation for its consistent dampening of IFN suppression across cell types.

### NS1 amino acid substitutions fine-tune polymerase activity and enhance replication in cell culture

**Fig 6:**
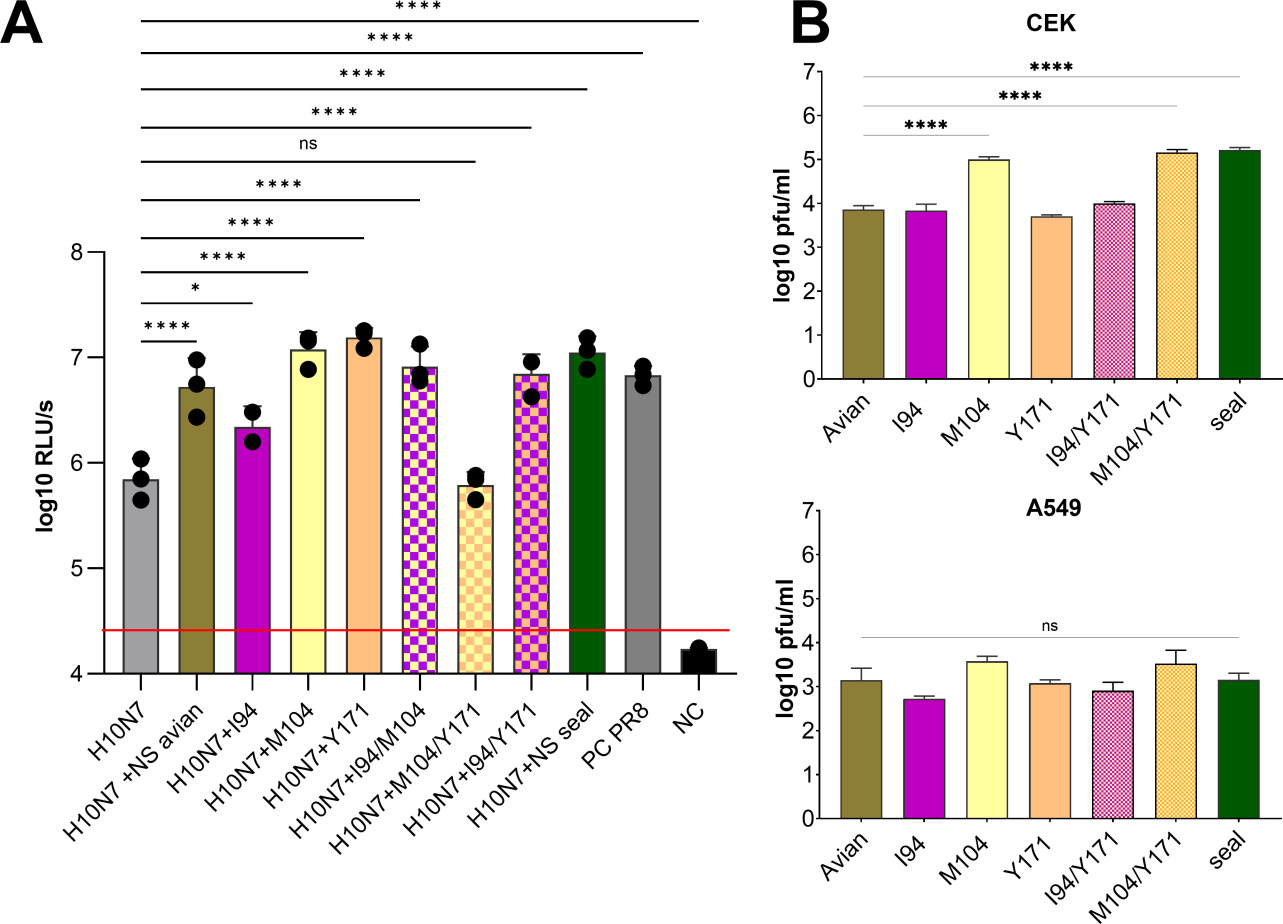
Impact of seal NS1 amino acid substitutions on polymerase activity and viral replication. HEK293T cells were transfected with pcDNA3 plasmids containing either avian or seal polymerase segments (PB2, PB1, and PA), NP, and NS1, including NS1 mutant versions with substitutions at residues 94, 104, and 171. Polymerase activity was evaluated using a minigenome reporter assay 24 h post-transfection. The assay was performed in triplicate and repeated three times, with results expressed as log_10_ relative light units (RLUs) of luciferase activity. Firefly-luciferase-expressing plasmid was used as a transfection control (**A**). Recombinant viruses carrying different NS1 amino acid substitutions (avian, single, or triple seal-NS1 amino acid substitutions) were generated using reverse genetics, with the avian H7N7 virus backbone. Attempts to generate a virus with double amino acid substitutions at residues 94 and 104 were unsuccessful. CEK and A549 cells were infected with the recombinant viruses, and viral replication was measured as plaque-forming units (log10 pfu/mL) 24 h post-infection by plaque assay in MDCK II cells. The experiments were conducted in triplicate and repeated three times, with results expressed as mean and standard deviation of plaque-forming units (log10 pfu/mL) (**B**). Asterisks indicate statistically significant differences (*P < 0.05, **P < 0.01, ***P < 0.001, and ****P < 0.0001). HEK293T, human embryonic kidney 293T cells; CEK, primary chicken embryonic kidney cells; A549, human lung adenocarcinoma cells; MDCK II, Madin-Darby canine kidney type II cells; NS1, non-structural protein 1; NP, nucleoprotein; PB2/PB1/PA, polymerase subunits; RLU, relative fluorescence units; pfu, plaque-forming units.

Given that NS1 amino acid substitutions affected host gene expression, we next asked whether these same amino acid substitutions also influence viral gene expression by modulating polymerase activity. While transcriptional shutoff broadly suppresses host gene expression, NS1 is also known to enhance viral polymerase activity, promoting efficient viral RNA synthesis and protein production (50). To test this, human cells were transfected with plasmids encoding the H10N7 viral polymerase complex along with wild-type or mutant NS1 proteins, including single and combined seal-derived substitutions (Fig. 6A). Both avian and seal NS1 significantly enhanced polymerase activity (*P* < 0.001), confirming a meticulous supportive role for NS1 amino acid substitutions in polymerase function. Amino acid substitution I94 alone produced the smallest, yet still significant, enhancement (*P* < 0.05). Interestingly, individual amino acid substitutions at residues 104 or 171 led to a significant increase in polymerase activity, but their combination abolished this enhancement (*P* > 0.05), suggesting a non-additive or antagonistic interaction. Notably, the addition of I94 to the M104 + Y171 combination, as in the seal NS1 triple mutant, restored polymerase-enhancing activity. These findings indicate that NS1 modulates host shutoff and viral polymerase activity through distinct but potentially interacting mechanisms, and specific combinations of amino acid substitutions can fine-tune these functions to optimize replication across different host environments.

Given that NS1 amino acid substitutions influenced IFN antagonism, host gene expression shutoff, and polymerase activity, we next assessed their impact on the multiple-cycle replication of the recombinant viruses carrying wild-type or mutant NS1 segments in both chicken and human cells at 24 h post-infection (Fig. 6B). In chicken cells, viruses expressing seal NS1, M104, or the M104 + Y171 combination showed significantly enhanced replication compared to the virus with wild-type avian NS1 (*P* < 0.0001). In contrast, in human cells, the same amino acid substitutions resulted in only a modest, statistically non-significant increase in viral replication, suggesting that additional viral genes may be required for efficient adaptation to human hosts.

Together, these results demonstrate that NS1 amino acid substitutions modulate multiple aspects of viral function and act synergistically to enhance replication efficiency in avian cells. Residue 104, in particular, appears to play a central role in driving polymerase activity and increasing replication.

### NS1 amino acid substitution signatures suggest host-specific co-evolution in seal H10N7 virus

**Fig 7:**
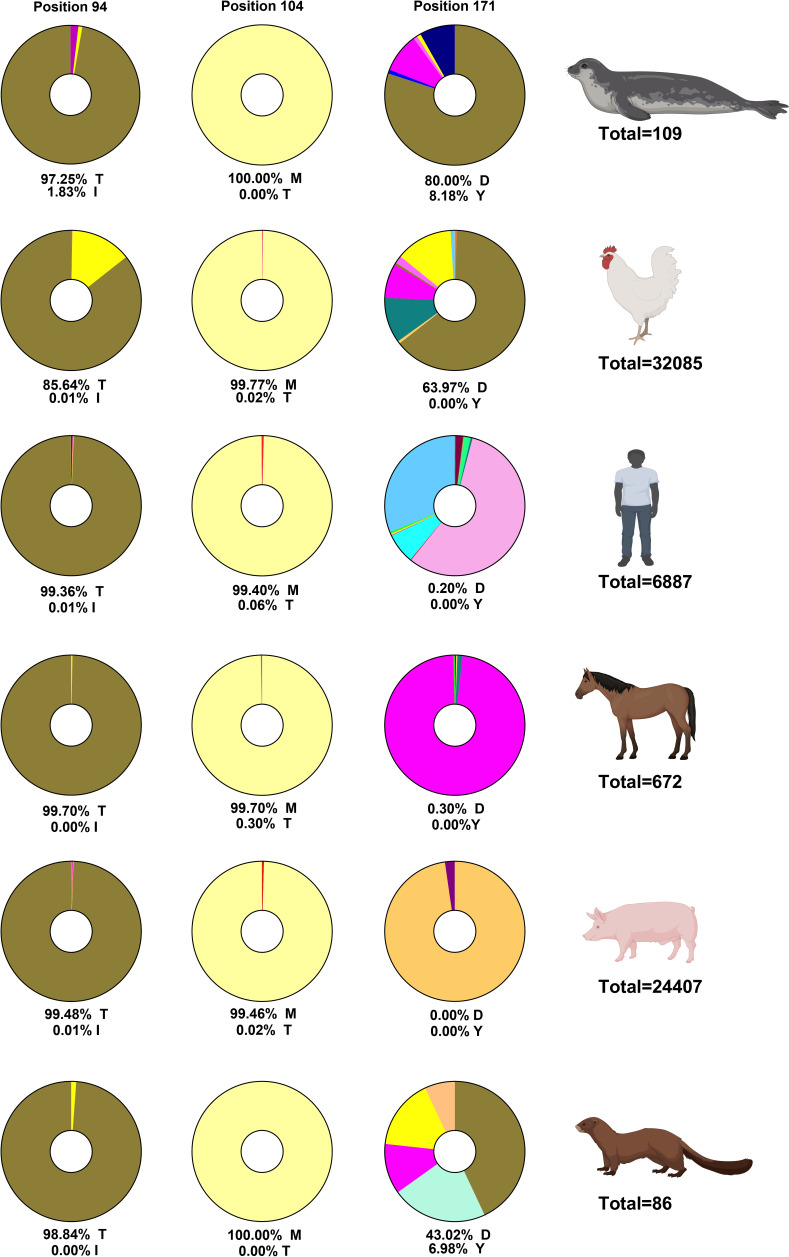
Prevalence of H10N7 NS1 amino acid substitutions in different hosts. The figure shows the prevalence of the key seal amino acid substitutions (T94, T104, and D171) in NS1 sequences from various host species, retrieved from GISAID (retrieval date: 23 August 2023). Complete NS1 protein sequences from different species were retrieved, aligned, and cleaned by removing ambiguous residues using MAFFT. The prevalence of amino acids at residues 94, 104, and 171 was estimated using Geneious. Seal NS1 of H10N7 carries T94, T104, and D171, while the closest avian ancestor H7N7 has I94, M104, and Y171. The figure was created using BioRender.

The synergistic yet balanced effects of the three NS1 amino acid substitutions, T94, T104, and D171 in the avian NS1 (compared to I94, M104, and Y171 in the seal NS1), on protein expression, stability, and function suggest a pattern of strict co-evolution. To further investigate this, we analyzed the prevalence of these polymorphisms in NS1 sequences from various hosts using data retrieved from GISAID (Fig. 7).

Among the three positions, residue 104 was the most conserved. The M104 variant in the seal virus was present in 99.4% of human and swine isolates, 99.7% of avian and equine isolates, and 100% of marine mammal and mink sequences. In contrast, the T104 variant of the avian H7N7 ancestor was extremely rare, suggesting that the change to M104 represents a reversion to the highly conserved, functionally favorable state.

Residue 171 showed greater host-associated variability. The avian-type D171 predominated in marine mammals (80%), birds (~64%), and mink (43%), whereas N171 dominated in equine (97.9%) and E171 in swine (97.7%). The Y171 variant in the seal virus, though uncommon overall, may represent an adaptive substitution that enhances viral fitness in marine mammals or compensates for the presence of the rare T104.

Residue 94 was less conserved than 104 but still showed a strong dominance of T94 across hosts: 97.3% in marine mammals, 85% in birds, 99.4% in humans, 99.7% in equine, 99.6% in swine, and 98.8% in mink. The I94 substitution in the seal virus is rare and may affect protein stability and function or act as a compensatory change linked to the unusual T104.

Taken together, these findings show that the seal H10N7 virus acquired three unique NS1 amino acid substitutions (I94, M104, and Y171) relative to its avian H7N7 ancestor. The replacement of the rare T104 with the highly conserved M104, together with co-occurring substitutions at positions 94 and 171, supports the hypothesis of host-specific co-evolution and functional adaptation of NS1 in marine mammals.

### Reversion to M104 highlights the co-evolutionary constraint on NS1 residue 104

**Fig 8:**
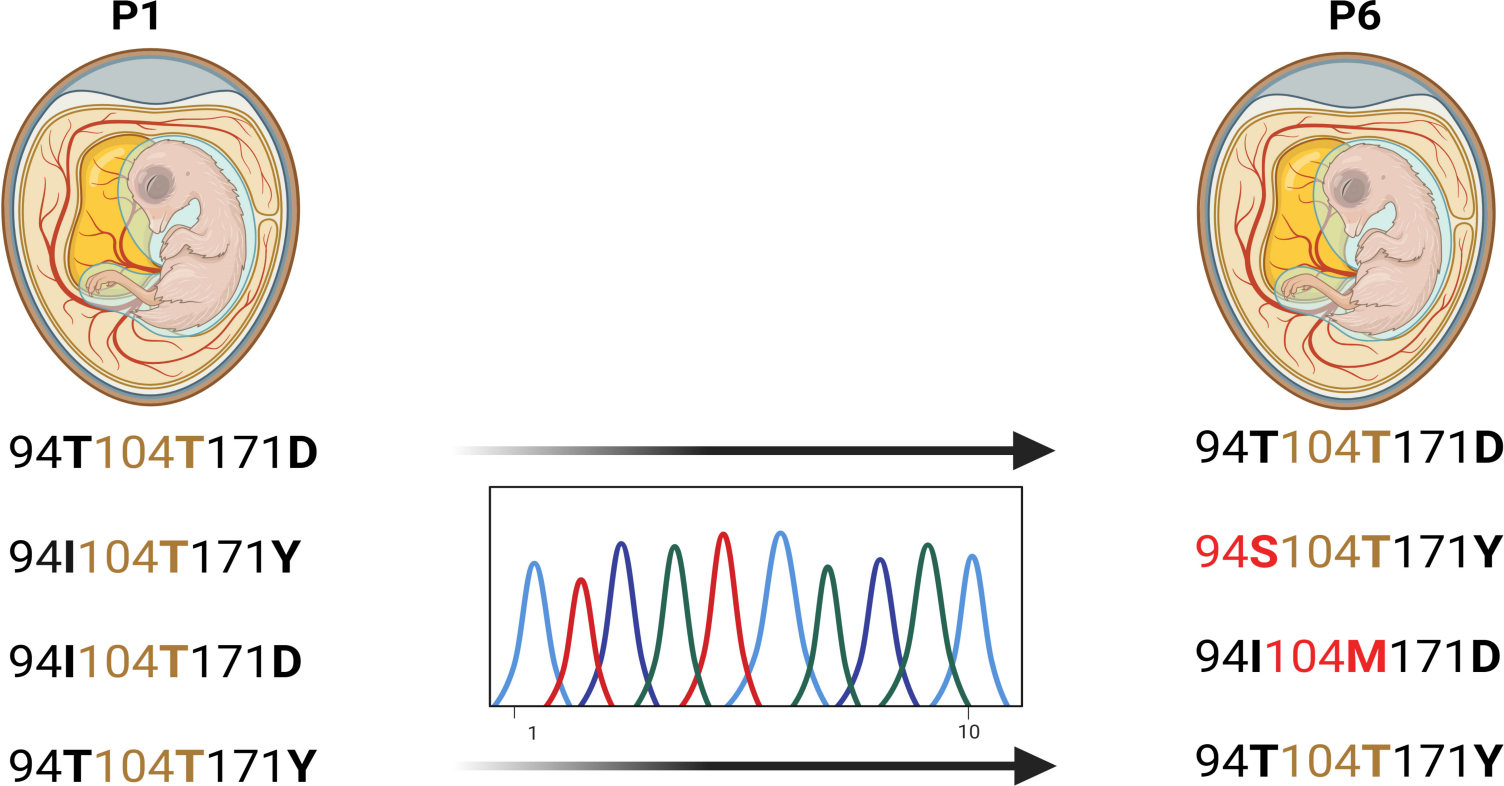
Amino acid substitution stability of NS1 residue 104 during serial egg passaging. Recombinant H7N7 avian viruses carrying the NS1 amino acid substitution T104, alone or in combination with amino acid substitutions at positions 94 and 171, were serially passaged in 14-day-old specific-pathogen-free chicken eggs for six consecutive passages (two eggs per passage per virus). Viral RNA was extracted from the allantoic fluid collected at passage 6, and the NS segment was amplified by RT-PCR. Sanger sequencing of the PCR products revealed that the virus harboring the I94, T104, and D171 amino acid substitutions reverted at position 104 from T104 to M104 after passaging, whereas viruses with other amino acid substitution combinations retained their original sequences at residue 104.

To test the hypothesis that residue 104 is part of a co-evolved amino acid substitution set, we serially passaged recombinant H7N7 viruses carrying the unique avian-type T104 in combination with polymorphisms at positions I/T94 or Y/D171 in embryonated chicken eggs (ECEs) for six consecutive passages (Fig. 8). The avian NS1 ancestral virus (T94/T104/D171) was used as a control. Viral RNA was extracted from the allantoic fluid, and the NS1 gene was sequenced via Sanger sequencing.

Intriguingly, the virus carrying the combination I94, T104, and D171 acquired a T104 to M104 reversion, resulting in the genotype I94/M104/D171. In contrast, viruses retaining T104 in the context of either T94 or Y171 maintained the original T104 residue throughout all passages (Fig. 8). These findings suggest that the rare T104 is only tolerated within a specific amino acid substitutional context, namely, with T94 and D/Y171, supporting the notion of strict co-evolution in the avian H7N7 ancestor. When this delicate balance is disrupted, as in the I94/T104/D171 combination, selective pressure favors reversion to the more stable and evolutionarily conserved M104.

## DISCUSSION

The increasing prevalence of AIVs in non-human mammals poses a serious risk for the emergence of zoonotic influenza viruses. Interestingly, although most seal-derived NS1 proteins suppressed IFN production as effectively as their avian precursors, the NS1 protein from seal H10N7 exhibited reduced IFN antagonism in avian cells but enhanced suppression in human cells (Fig. 4B). Surprisingly, this reduction in IFN suppression in chicken cells correlated with increased viral replication (Fig. 6B), indicating that NS1 activity may shift during host adaptation and that IFN antagonism can be fine-tuned in a host-specific manner rather than universally maximized. This observation aligns with earlier reports that avian influenza viruses are restricted in human cells by type I IFN responses, even though their NS1 proteins counteract IFN induction as effectively as those of human strains. Conversely, some studies have found no direct correlation between IFN-β induction and replication of AIVs in human A549 cells (51).

One particularly striking observation in our study was that seal-derived H10N7 NS1 proteins were more stable than their avian counterparts, despite being expressed at similar levels. This increased stability may contribute to the functional tuning of NS1 activity during host adaptation. Previous studies have demonstrated that NS1 stability is crucial for efficient IFN antagonism, as amino acid substitutions reducing NS1 half-life (e.g., I178V in H7N9) impair its ability to suppress IFN responses (52). NS1 must remain stable long enough to interact with host factors like CPSF30, critical for IFN inhibition (40, 53). Post-translational modifications, such as acetylation, also modulate NS1 function and stability, influencing viral replication and immune evasion (54). Our observation that seal H10N7 NS1 shows altered IFN suppression despite similar expression levels likely reflects subtle effects on protein stability or host interactions rather than abundance. These findings support a model where NS1 amino acid substitutions fine-tune IFN antagonism by affecting stability and function to optimize replication.

Although residues 94, 104, and 171 are located in the well-characterized NS1 effector domain, their roles in IAV replication and host interactions remain largely unexplored. We found that residue 94 increased NS1 expression, abolished host transcription shutoff, and reduced IFN antagonism across hosts. When combined with residue 171, it also impairs virus rescue. In contrast, M104 is highly conserved, enhances NS1 stability, increases polymerase activity, and improves IFN suppression across hosts. Residue 171 displayed species-specific variation and influenced NS1 function in a similar way to M104. Previous studies have shown that residue 171 differs between seasonal and pandemic 2009 H1N1 strains. Introducing A171Y in the PR8 background increased NS1 abundance, suppressed IFN and IFN-stimulated gene expression in human lung cells, and altered viral titers (55). Another study identified residue 171 as one of the most frequently mutated NS1 positions in human IAVs from 1918 to 2004, with the bulky tyrosine (Y171) contributing to stabilization of NS1 conformation (56). Unfortunately, all attempts to study interactions between seal and avian NS1 proteins and host factors, namely CPSF30 and eIF4G, were unsuccessful. This was likely due to global transcriptional shutoff in cell-based systems or unfavorable stability conditions in cell-free systems.

Last but not least, the impact of NS1 expression on viral polymerase activity in the minigenome assay warrants further investigation to elucidate the underlying mechanisms. This influence may not be strictly linked to NS1 abundance, as seen with the minimal effect of I94, nor solely to protein stability, given the comparable enhancing effects observed with other amino acid substitutions, particularly when combined in avian or seal NS1. Previous studies have shown that NS1 of PR8 can enhance polymerase activity both directly, through interactions with host proteins that facilitate RNA synthesis, and indirectly, by suppressing host mRNA processing or antiviral factors to favor viral protein expression and replication (50, 57, 58).

Together, our study highlights that avian influenza viruses circulating in seals carry NS1 proteins derived from avian ancestors, with amino acid substitutions suggesting host-specific adaptation. Despite sequence variation, NS1 structural integrity and expression were largely preserved across avian, seal, and human cells, emphasizing its essential role in replication and host adaptation. Seal H10N7 NS1 showed altered IFN antagonism and increased protein stability, indicating fine-tuning of function rather than maximal suppression. Residues 94, 104, and 171 contributed to these functional differences, with M104 enhancing stability and polymerase activity. Overall, our findings underscore the importance of monitoring NS1 evolution in non-human mammals, as such adaptations may influence viral fitness and zoonotic potential, reinforcing a One Health perspective.

## MATERIALS AND METHODS

### Biosafety

The generation and characterization of recombinant viruses were conducted at Institute of Molecular Virology and Cell Biology, Friedrich-Loeffler-Institut (FLI), Federal Research Institute for Animal Health, Greifswald-Insel Riems, Germany

### Sequence analysis and modeling

NS1 sequences of AIVs from seals (1980–2023) were compared to their closest avian ancestors (Table 1). Additional NS1 sequences from influenza A viruses isolated from marine mammals, birds, humans, minks, horses, and swine were retrieved from GISAID (retrieval date: 23 August 2023). Sequences were aligned using Geneious Prime (v2021.0.1) with the MAFFT algorithm, and ambiguous residues were removed. The prevalence of amino acids at positions 94, 104, and 171 was calculated in Geneious. Midpoint-rooted phylogenetic trees were constructed with IQ-TREE, and representative sequences were selected to generate the final tree using phylogeny.fr and edited in FigTree. NS1 tertiary dimer structures were predicted with SWISS-MODEL and further refined in Geneious.

### Plasmids

pHW2000, pCAGGS, and pcDNA3 plasmids carrying the NS inserts of the indicated viruses were generated in this study. The NS segment was amplified using the Omniskript RT Kit (Qiagen, Germany) and Phusion High-Fidelity DNA Polymerase (New England Biolabs, USA). PCR products were purified on 1% agarose gel and extracted using the Hi Yield Gel/PCR DNA Fragment Extraction Kit (SLG, Germany). The competent *E. coli* XL-1 blue strain was transformed, and plasmids were isolated from bacterial cultures with the Plasmid Midi Kit (Qiagen, Germany). After the initial plasmid generation, site-directed mutagenesis with custom primers and QuikChange II Site-Directed Mutagenesis Kit (Agilent, USA) was performed to generate single and double mutants. Sequencing of all the plasmids was performed by Eurofins or Microsynth (Germany).

### Cells

Human embryonic kidney cells 293T (HEK293T), human lung adenocarcinoma (A549), Madin-Darby canine kidney type II (MDCK II), and chicken fibroblast (DF1) cell lines were obtained from the Cell Repository of FLI. Cell lines of the kidney (SEK2b) of a harbor seal (*Phoca vitulina*) were kindly provided by Dr. Matthias Koenig from Seltersberg Biomedical Research Center (BFS) Giessen, Germany. Primary chicken embryonic kidney cells (CEK) were prepared from the kidneys of 18-day-old chicken embryos (59).

### Viruses

Recombinant viruses were generated as previously described (60). For the generation of viruses, the PB2, PB1, PA, HA, NP, NA, and M segments of A/turkey/Germany/R534/2013 (H7N7) were used in addition to the corresponding NS mutants of A/harbor seal/Germany/1/2014 (H10N7). Briefly, 75% confluent HEK293T cells were co-transfected with 0.250 μg of each of the eight plasmids by using Lipofectamine 2000 reagent (Invitrogen, USA). The supernatant was collected after 48 h of transfection and subsequently inoculated into 11-day-old embryonated chicken eggs (ECEs) obtained from specific-pathogen-free (SPF) chickens (VALO BioMedia GmbH, Germany). After 48–72 h of incubation, allantoic fluid containing the virus was harvested and stored at −80°C. Successful virus generation was confirmed by Sanger sequencing.

### Western blot

Protein expression in different cells was analyzed by Western blotting as previously described (49). Preliminary experiments were performed to optimize plasmid concentration, transfection reagents, and post-transfection incubation time for NS1 expression. Following optimization, cells were transfected with 2 µg of NS1 plasmids using Opti-MEM with GlutaMAX and Lipofectamine 2000 (Fisher Scientific, Germany). Transfected cells were incubated at 37°C with 5% CO_2_ for 24 h, harvested, washed twice with PBS, and centrifuged at 13,000 rpm for 10 min. Cell pellets were resuspended in Laemmli buffer (1:1 with PBS) and heated at 95°C for 5 min. Proteins were separated on 10% SDS-PAGE, transferred to nitrocellulose membranes, and blocked with 5% low-fat milk in TBS-T for 1 h at room temperature. Primary antibodies used were rabbit anti-NS1 (produced in-house) and monoclonal anti-β-actin (Abcam, UK) or -GAPDH (Abcam, UK), followed by IRDye 680/800CW (Li Cor, USA) secondary antibodies. Signals were detected using an Odyssey Clx Imager. Band intensities were quantified using Image Studio Lite 5.2, and data are expressed as mean ± SD of the relative NS1/housekeeping ratio.

### Luciferase reporter assay

To evaluate NS1-mediated inhibition of the IFN-I pathway, luciferase reporter assays were performed as previously described (47). DF1 and SEK2b cells were seeded in 6-well plates and transfected with a plasmid mixture containing: 0.5 µg Firefly luciferase (FFL) reporter plasmid (human or chicken p125:IFN-β-Pro-FFL), 0.005 µg pCMV-Renilla (for normalization), 0.2 µg human or chicken pMDA5-Δ (trigger plasmid), and 0.5 µg pcDNA3 encoding NS1 or empty vector. For transcription shutoff analyses, 0.25 μg pCAGGS-FFL reporter plasmid was cotransfected with the NS1 coding plasmids. Transfections were performed using Lipofectamine 2000 (Thermo Fisher, USA) following the manufacturer’s instructions. At 20 h post-transfection, cell lysates were collected, and luciferase activity was measured using the Dual-Luciferase Reporter Assay System (Promega, USA). Firefly and Renilla signals were quantified with a Centro LB963 microplate luminometer (Berthold, Germany). Experiments were conducted in triplicate, and results are reported as normalized means ± standard deviations (SDs).

### Protein stability assay

Protein stability was assessed using a modified version of a previously described method (61). Briefly, MDCK II cells were infected with the viruses at a multiplicity of infection (MOI) of 0.01 for 1 h with the addition of trypsin. At 24 h post-infection, cycloheximide (CHX) in DMSO was added to a final concentration of 100 µg/mL to halt protein synthesis. Cells were harvested at 0, 8, 12, 24, and 32 h after CHX addition and processed following the standard Western blot sample preparation protocol. NS1 protein levels were quantified using the Odyssey CLx Imager and Image Studio software. NS1 band intensities were normalized to the corresponding β-actin loading control for each sample. Normalized NS1 values from three replicates were averaged, and the mean value at 0 h was set to 100% for each virus. The half-life (*t*_1/2_) of NS1 was determined from the first-order decay of normalized protein levels using the equation *t*_1/2_ = ln2/*k*, where *k* is the decay constant derived from fitting NS1 remaining (%) versus time to an exponential decay model. Fold changes in stability were calculated by dividing the mutant NS1 half-life by the avian NS1 half-life. The recombinant H7N7 viruses did not replicate efficiently in seal cells; therefore, we did the infection experiments in MDCK II cells.

### Legendplex assay

LEGENDplex Human Anti-Virus Response Panel 1 (BioLegend, Germany) was used to quantify Type I and III IFN in A549 cells as previously conducted (47). Briefly, cells were infected with the indicated recombinant viruses in the presence of trypsin for 24 h, followed by stimulation with poly(I:C) for an additional 24 h. Supernatants were collected and analyzed using this bead-based multiplex immunoassay according to the manufacturer’s protocol. Data acquisition was performed by flow cytometry, and results were analyzed using the LEGENDplex Data Analysis Software (47).

### Inhibition of human gene expression

To assess the effect of NS1 proteins on host gene expression, HEK293T cells were seeded in 24-well plates (2.5 × 10^5^ cells/well, triplicate) and transiently co-transfected using Lipofectamine 3000 (Invitrogen) with 1 μg/well of pCAGGS-HA-NH2 NS1 expression plasmids or empty pCAGGS-HA-NH2 as a control. Co-transfections included 25 ng/well of pCAGGS plasmids encoding Gaussia luciferase (Gluc) (62) and the green fluorescent protein (GFP) (63). Cells were incubated at 37°C, and at 6 h post-transfection, the medium was replaced, followed by an additional 24 h incubation. GFP expression was assessed by fluorescence microscopy (Olympus IX81) and photographed using a QIMAGING Retiga 2000R camera. Gluc activity in the tissue culture supernatants was measured using Biolux Gaussia luciferase reagent (New England Biolabs) and quantified with a Lumicount luminometer (Packard). Mean values and standard deviations (SDs) were calculated using Microsoft Excel.

### Viral polymerase activity assay

The influence of NS1 amino acid substitutions on viral polymerase activity was assessed using a luciferase-based minigenome assay as previously described (64). Sub-confluent HEK293T cells were transfected with pCAGGS plasmids encoding the polymerase complex segments (PB1, PB2, PA, and NP) together with different NS1 mutants, H7-NanoLuciferase (NanoLuc), and Firefly luciferase. As a negative control, PB2 was replaced with an empty pCAGGS vector. At 24 h post-transfection, cells were lysed using Lysis-Juice (PJK, Germany). Firefly and NanoLuc substrates were added, and luminescence was measured using a GloMax Discover Microplate Reader. NanoLuc signals were normalized to Firefly luciferase activity.

### Viral replication and titration

Recombinant viruses were used to infect CEK or A549 cells at an MOI of 0.001 for 1 h. After infection, cells were washed with PBS and overlaid with appropriate minimal essential medium supplemented with bovine serum albumin (BSA). Plates were incubated at 37°C with 5% CO_2_ for 24 h. Cells and supernatants were collected and stored at –80°C prior to titration. Virus titers were determined as plaque-forming units per milliliter (PFU/ml). Tenfold serial dilutions were added to confluent MDCK II monolayers in 12-well plates for 1 h at 37°C and 5% CO_2_. After washing twice with PBS, cells were overlaid with 0.9% Bacto Agar/MEM containing 0.2% BSA and incubated for 72 h. Plates were fixed with 0.1% crystal violet in 10% formaldehyde, and plaques were counted under a light microscope.

### Serial egg passaging and NS1 sequencing

Recombinant H7N7 avian viruses carrying NS1 amino acid substitutions (T104 alone or combined with I94 and D171) were serially passaged in 14-day-old SPF chicken eggs for six consecutive passages (two eggs per passage per virus). Viral RNA was extracted from allantoic fluid at passage 6, and the NS segment was amplified by RT-PCR. Sanger sequencing of the PCR products was performed to assess the stability of residue 104.

### Statistical analysis

All data were analyzed using GraphPad Prism (version 10.2.1, GraphPad Software, San Diego, CA, USA). Results are presented as mean ± standard error of the mean unless otherwise indicated. Statistical significance for multiple group comparisons was calculated using one-way or two-way ANOVA, followed by the Kruskal–Wallis test as appropriate. Statistical significance for NS1 half-lives was evaluated using an unpaired *t*-test. A *P*-value < 0.05 was considered statistically significant. Western blot data for protein expression quantification were normalized to the corresponding loading control before statistical evaluation.

## Data Availability

All data generated or analyzed during this study are included in the published article. No new data sets were generated or deposited in public repositories.
